# Influencing factors of good quality of life among chronic obstructive pulmonary disease patients living in Zhejiang Province, China

**DOI:** 10.1038/s41598-024-59289-9

**Published:** 2024-04-15

**Authors:** Yubing Ying, Siriyaporn Khunthason, Tawatchai Apidechkul, Kessarawan Nilvarangkul

**Affiliations:** 1 School of Health Science, Mae Fah Luang University, 333 Moo 1, Ta Sud Subdistrict, Muang District, 57100 Chiang Rai Province Thailand; 2 Chiang Rai Rajabhat University, 80 Moo 9 Phaholyothin Road, Muang District, 57100 Chiang Rai Thailand

**Keywords:** COPD, Associated factors, SGRQ, Good QOL, Diseases, Health care, Risk factors

## Abstract

Chronic obstructive pulmonary disease (COPD) is a chronic, progressive and debilitating disease that affects quality of life (QOL), especially among patients living in poor environments. This study aimed to determine the influencing factors of good QOL among COPD patients living in Zhejiang, China. A cross-sectional study was conducted to collect data from participants in six tertiary hospitals in Zhejiang Province by a simple random sampling method. A validated questionnaire was used to collect general information, environmental factors, and COPD stage. The standardized St. George's Respiratory Questionnaire (SGRQ) was used to assess QOL. Logistic regression was used to determine influencing factors of good QOL among COPD patients at a significance level of α = 0.05. A total of 420 participants were recruited for analysis. The overall prevalence of patients with good QOL was 25.7%. Six variables were found to be associated with good QOL in the multivariable analysis. Patients who were employed had 2.35 times (95% CI 1.03–5.34) greater odds of having good QOL than those who were unemployed. Those whose family income was higher than 100,000 CNY had 2.49 times (95% CI 1.15–5.39) greater odds of having good QOL than those whose family income was lower than 100,000 CNY. Those who had treatment expenses less than 5,000 CNY had 4.57 (95% CI 1.57–13.30) times greater odds of having good QOL than those who had treatment expenses of 5,000 CNY or higher. Those who had mild or moderate airflow limitation were 5.27 times (95% CI 1.61–17.26) more likely to have good QOL than those who were in a severe or very severe stage of COPD. Those who had a duration of illness less than 60 months had 5.57 times (95% CI 1.40–22.12) greater odds of having good QOL than those who had a duration of illness of 120 months or more. Those who were not hospitalized within the past 3 months had 9.39 times (95% CI 1.62–54.43) greater odds of having good QOL than those who were hospitalized more than twice over the past 3 months. Socioeconomic status, disease stage and accessibility were associated with good QOL among COPD patients in Zhejiang Province, China. Increasing family income and implementing measures to improve the accessibility of medical care, including developing a proper system to decrease the cost of treatment for COPD patients, can improve patients’ QOL.

## Introduction

Chronic obstructive pulmonary disease (COPD) is a substantial public health concern worldwide^1^ and is an irreversible progressive disease characterized by persistent airflow limitation^2^. COPD has been identified as the fourth leading cause of death worldwide and is projected to be the third leading cause of death in middle-income countries by 2030^3^. COPD is also defined as a disease with one of the largest health economic burdens in many countries, including China^4^. The Chinese government spent more than $73 billion caring for and treating patients with several chronic diseases, including COPD^5^. Moreover, the prevalence of COPD among Chinese individuals aged 40 years or older increased from 7.6% in 2009 to 13.7% in 2018^6,7^. The mortality rate was 79.4 per 100,000 population in 2013, which was higher than the global mortality rate (50.7 per 100,000 people) in the same year^8^. In the context of an aging society, including economic development based on industry, many people will suffer from COPD in China^9^.

The main problem for COPD patients is quality of life (QOL)^10^. Several factors contribute to the level of QOL among COPD patients, including its pathogenesis according to patients’ traits, socioeconomic status, and family support. Most COPD patients often suffer from poor QOL beyond suffering from its pathogenesis, such as symptoms and limited medical access^9,11–13^. A large proportion of COPD research conducted in China focuses on treatment and care, while a few publications emphasize the improvement in QOL in these populations by using the St. George's Respiratory Questionnaire (SGRQ)^14,15^. However, the QOL of COPD patients should be one of the most important issues for public health, particularly during the pandemic of COVID-19 due to both diseases’ impact on human lungs^16^. The suffering of COPD patients in China increased because of the incomplete function of healthcare services in China, ultimately leading to poor QOL.

COPD prevalence varies across different areas in China. The prevalence of COPD in Zhejiang Province, which is one of the largest, high-density, and industrial areas, ranges between 12.8 and 14.5% among people aged 40 or older^17,18^. COPD was reported as the main public health problem in Zhejiang Province, and its healthcare services system was the most highly impacted in China^6,7,18,19^. Zhejiang Province is the third largest industrial province in China with high levels of industrial emissions^19^. A large proportion of people aged 40 years and over smoke, and the environment has very poor air quality^20,21^, including exposure to outdoor air pollution from industrial factories and agricultural burning by residents; COPD patients face a problem regarding QOL^22,23^. Nevertheless, there is no scientific information about QOL among COPD patients living in areas with poor air pollution, such as Zhejiang Province, China. Therefore, this study aimed to assess QOL and determine the factors associated with good QOL among COPD patients in Zhejiang Province, China.

## Methods

### Study design and setting

An analytical cross-sectional study was used to assess the level of QOL among COPD patients and determine the factors associated with good QOL among COPD patients in the respiratory departments of six tertiary hospitals in Zhejiang Province, Southeast China.

### Study population and study sample

The study population was COPD patients who attended one of six hospitals in Zhejiang Province, China. The inclusion criteria were those aged 40 years and over and who were diagnosed with COPD by a physician. However, those who had been diagnosed with lung cancer, bronchiectasis, pneumoconiosis, or other restrictive lung ventilation dysfunction were excluded from this study.

The sample size was calculated by the standard formula for a cross-sectional study^24^, n = [Z^2^_α/2_ PQ]/e^2^, where $Z$ = the value of the standard normal distribution corresponding to the desired confidence level ($Z=$ 1.96 for 95% CI); P is the prevalence of good QOL among COPD patients in China at 14.1%, which was 0.14^25^; Q is the difference of one and P (1-P); and e is the desired precision (0.05 or 5%). Allowing for 15% error throughout the study process, at least 213 individuals were required for the analysis.

**Figure 1:**
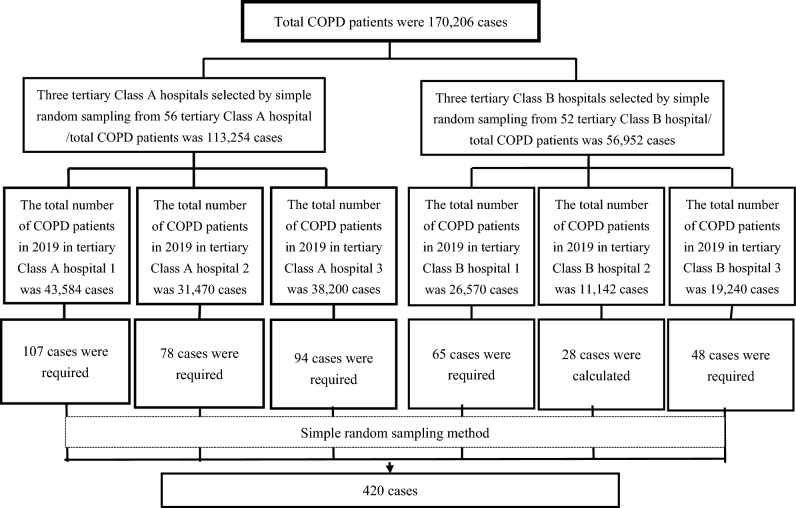
Flow of the study samples selection from six selected hospitals.

After the sample size was calculated, six tertiary hospitals were randomly selected from among 108 hospitals by a computer-generated randomization method, as shown in the following flowchart (Fig. 1). To ensure that each sample site had an equal probability of providing a sample, simple random sampling with a proportional allocation of 0.25% was used to select the sample at each study site. Those who were selected were screened according to the inclusion and exclusion criteria before the initiation of data collection.

### Research instruments

A validated questionnaire and the standardized SGRQ^26^ were used for data collection. The validated questionnaire was developed according to the relevant literature and guidelines and discussed with experts in the field. The questionnaire consisted of three parts: general information about the participants, environmental factors, and the stage and treatment of COPD. In part one, twelve questions were used to collect general information about the participants, such as age, sex, marital status, educational level, occupational status, and household income. In part two, five questions were used to collect information on environmental factors such as residence, distance to the hospital, and hospital transport method. In the last part, five questions were used to collect information on the COPD stage and treatment of the participants.

The standardized SGRQ^26^ was used to assess QOL among the participants. The SGRQ contains 50 items for assessing QOL in 3 domains: symptoms, activity, and impact. Each questionnaire response has a unique empirically derived ‘weight’^26^. SGRQ scores range from “0” to “100”, and “0” represents the best QOL. Finally, the participants were divided into three levels of QOL according to their scores: poor (scores of 66.68–100.00), moderate (scores of 33.34–66.67), and good (scores of 0.00–33.33)^27^. The Mandarin Chinese version of the SGRQ was provided by St. George’s, University of London, and had been tested as a valid and responsive tool for assessing the QOL of Chinese COPD patients in China^28^.

### Research instrument development

All questions were examined for validity and reliability by the item-objective congruence (IOC) method^29^ and verified by three external experts in the field: a medical doctor, an epidemiological expert, and a nurse. Each expert evaluated each item, and the scores ranged from − 1 to + 1 (if a question complied with the study scope and objective, it was scored + 1, suspicious = 0, and inconsistent = − 1). For the evaluation results, questions that scored an average score ≥ 0.70 were included in the questionnaire. The questions with average scores between 0.51 and 0.70 were modified before being included in the questionnaire. Questions with an average score below 0.5 were excluded from the questionnaire.

Before data collection, a pilot test was conducted at two selected tertiary hospitals in Zhejiang Province with 30 samples (15 samples from each) who had similar characteristics to the study sample. Only the questions with Cronbach’s alpha value ≥ 0.75 were included in the questionnaire. Finally, all the questions were reviewed by the research team before the data collection began.

### Measures

Body mass index (BMI) was classified into three categories: less than 18.50 (underweight), 18.5–23.9 (normal weight), and ≤ 24.0 (overweight)^30^. The airflow limitation severity in COPD was classified into four levels: mild (forced expiratory volume in one second (FEV1) ≥ 80% of predicted), moderate (50% predicted ≤ FEV1 < 80% predicted), severe (30% predicted ≤ FEV1 < 50% predicted) and very severe (FEV1 < 30% predicted)^2^. The QOL among COPD patients was classified by the standardized SGRQ into three levels: poor, moderate, and good.

### Data collection procedure

Six tertiary hospitals from among 108 hospitals with respiratory departments listed by the Ministry of Health and Population were randomly selected by a computer-generated randomization method and divided into two categories: tertiary class A hospitals and tertiary class B hospitals. Permission to assess the hospital was granted by the department director after sending official letters. The respiratory department staff were contacted to explain the purpose and questionnaire again to obtain their agreement to collect data in both outpatient and inpatient departments.

The purpose of this study and the content of the questionnaire were explained to the selected participants. Afterward, a written informed consent form was signed before starting data collection. The questionnaires were completed by the researcher. One hundred ninety-one participants were interviewed face-to-face, and 229 participants were interviewed by telephone due to coronavirus disease 2019 (COVID-19). Each face-to-face interview took 30 min, and each telephone interview took 40 min. Before ending the interview, completed questionnaires were checked again to ensure that there were no missing data. Data were collected between October and December 2021.

### Statistical analysis

Data were entered into the spreadsheet and checked for any errors before being imported into the SPSS program (Version 24, Chicago, IL). Continuous data were analyzed and are presented as frequencies, means, maximums, minimums, and standard deviations to describe the participants’ characteristics. Categorical data are presented as percentages. QOL was divided into three levels: low, moderate, and high.

A chi-square test and Fisher’s exact test were used to preliminarily test the associations between factors and good QOL. Logistic regression was used to determine the associations of factors and good QOL at a significance level α = 0.05 in both univariate and multivariate analyses. In the multivariate analysis, all predicted variables were entered into the model before non-statistical variable(s) were considered to exclude from the model. In this step, one most non-statistical variable was excluded from the model first, and considered the fit of the model by using the Hosmer–Lemeshow chi-square test before excluding the remaining non-statistical variable(s) in the model. The Cox-Snell R^2^ and Nagelkerke R^2^ were used to determine the fitness of the model before interpreting the final model.

### Ethics approval and consent to participate

Ethical approval was obtained from Mae Fah Luang University (No. EC 21073-18). Participants were recruited on a voluntary basis. On the date of data collection, each participant received all the necessary information about the study protocol, the purpose of the survey and the potential risks. Participants were asked to sign consent forms before starting the interview. The study procedures were performed in accordance with the relevant guidelines, regulations, and within the Declaration of Helsinki of 1975, as revised in 2000 (5).

## Results

A total of 420 COPD patients were recruited into this analysis: 56.4% were males, 48.1% were aged 70 years and over, and 67.1% were married. Approximately one-fifth (21.2%) were illiterate, 73.3% were unemployed, and 57.9% had an annual family income of less than 100,000 CNY ($14,750). Approximately twenty percent of participants (19.2%) faced problems with medical expenses, 69.8.0% self-paid for medical expenses, 73.3% had comorbidities, and 18.3% were current smokers (Table 1).

**Table 1:** General information of participants (n = 420).

Characteristics	n	%
Sex		
Male	237	56.4
Female	183	43.6
Age (years)		
< 60	105	25.0
60–70	113	26.9
> 70	202	48.1
*Min* = *40, Max* = *92, Mean* = *69.2, SD* = *12.5*		
Marital status		
Married	282	67.1
Single	18	4.3
Divorced or widowed	120	28.6
Education		
Illiterate	89	21.2
Primary	129	30.7
Secondary	127	30.2
Postsecondary	75	17.9
Occupational status		
Unemployed	308	73.3
Employed	112	26.7
Family annual income in CNY (1UDS = 6.78 CNY)		
< 100,000	243	57.9
≥ 100,000	177	42.1
*Median* = *77,000, IQR* = *39,500*		
Cost of COPD treatment expenses per year in CNY		
< 5000	247	58.8
> 5000	173	41.2
Person who supports medical expenses		
Family members	98	23.3
Participant	293	69.8
Others	29	6.9
Having problems on medical expenses		
Yes	81	19.3
No	339	80.7
BMI		
Normal weight	238	56.7
Underweight	79	18.8
Overweight	103	24.5
Type of medical insurance		
Employees	127	30.2
Basic medical insurance for urban and rural residents	293	69.8
Having comorbidities		
No	112	26.7
Yes	308	73.3
Having 1–2 diseases	254	60.5
Having 3–4 diseases	54	12.9
Types of comorbidities		
Hypertension	201	47.9
Diabetes	118	28.1
Osteoporosis	53	12.6
Others	156	37.1
Current cigarette smoking behaviour		
Current smoker	77	18.3
Ex-smoker of cigarettes	176	41.9
Non-smoker of cigarettes	167	39.8
Duration of smoking (months)		
< 60	11	2.62
61–120	7	1.7
121–360	95	22.6
> 360	140	33.3
Number of cigarettes smoked per day		
< 15	231	55.0
15–30	152	36.2
> 30	37	8.8

Nearly half of the participants (47.6%) lived in rural areas, and 48.3% went to a hospital by themselves. Half of the participants (43.8.0%) lived with others, and 40.5% had experienced exposure to secondhand smoke (Table 2).

**Table 2:** Environmental factors of participants (n = 420).

Characteristics	n	%
Area of residence		
Rural	200	47.6
Urban	220	52.4
Distance from residence to hospital (km)		
< 10	232	55.2
10–30	188	44.8
A person who took to a hospital		
Self	203	48.3
Family members	200	47.6
Others	17	4.0
Living with		
Alone	236	56.2
Living with other	184	43.8
History of exposing to the second-hand smoke		
Yes	170	40.5
No	250	59.5

Almost half (49.5%) had severe airflow limitation. A large proportion (61.9%) were reported to have been diagnosed with COPD for 60 months or more, 44.8% did not have home oxygen therapy available, and 49% had been hospitalized at least once in the past three months. A large proportion (62.9%) had visited their doctor 5 times or more in the last three months. Only one-fourth (25.7%) of the participants had good QOL (Table 3).

**Table 3:** Characteristics of COPD and QOL (n = 420).

Characteristics	n	%
Airflow limitation severity		
Mild to moderate	212	50.5
Severe to very severe	208	49.5
Length of being COPD diagnosed (months)		
< 60	160	38.1
60–120	92	21.9
> 120	168	40.0
Having home oxygen therapy		
No	188	44.8
Short term	164	39.0
Long-term	68	16.2
Hospitalized day within the past 3 months		
None	214	51.0
1	83	19.8
> 2	123	29.2
Visit a doctor within the past 3 months		
< 5	156	37.1
5–10	162	38.6
> 10	102	24.3
Levels of QOL		
Poor	104	24.8
Moderate	208	49.5
Good	108	25.7

In the univariable analysis, 16 variables were found to be associated with good QOL: age, annual family income, cost of COPD treatment, BMI, medical insurance, number of comorbidities and types of comorbidities, such as hypertension, diabetes, and osteoporosis, current cigarette smoking, duration of smoking, distance from residence to hospital, exposure to secondhand smoke, airflow limitation severity, duration of illness, home oxygen therapy, number of hospitalizations within the past 3 months and doctor visits within the past 3 months. Other variables were not found to be associated with QOL (Table 4).

**Table 4:** Factors associated with good-QOL in univariate and multivariate analyses (n = 420).

Factors	Good QOL		Poor to moderate QOL		OR	95% CI	p value	AOR	95% CI	p-value
	n	%	n	%						
Sex										
Male	56	23.6	181	76.4	1.00					
Female	52	28.4	131	71.6	1.28	0.83–1.99	0.266			
Age (years)										
< 60	61	58.1	44	41.9	18.62	9.55–36.28	< 0.001*			
60–70	33	29.2	80	70.8	5.54	2.81–10.91	< 0.001*			
> 70	14	6.9	188	93.1	1.00					
Marital status										
Married	77	27.3	205	72.7	1.50	0.90–2.52	0.12			
Single	7	38.9	11	61.1	2.55	0.89–7.26	0.08			
Divorced or widowed	24	20.0	96	51.2	80.0					
Education										
Illiterate	21	23.6	68	76.4	0.90	0.47–1.71	0.743			
Primary	28	21.7	101	78.3	1.33	0.72–2.48	0.367			
Secondary	37	29.1	90	70.9	1.34	0.67–2.70	0.406			
Postsecondary	22	29.3	53	70.7	1.00					
Occupational status										
Unemployed	76	24.7	232	75.3	1.00			1.00		
Employed	32	28.6	80	71.4	1.22	0.75–1.98	0.42	2.35	1.03–5.34	0.042*
Family annual income in CNY										
< 100,000	32	13.2	211	86.8	1.00			1.00		
> 100,000	76	42.9	101	57.1	4.96	3.08–7.99	< 0.001*	2.49	1.15–5.39	0.02*
Cost of COPD treatment per year in CNY										
< 5,000	102	41.3	145	58.7	19.58	8.34–45.94	< 0.001*	4.57	1.57–13.30	0.005*
> 5,000	6	3.5	167	96.5	1.00			1.00		
Person who supports medical expenses										
Myself	74	25.3	219	74.7	1.00					
Family members	23	23.5	75	76.5	0.91	0.53–1.55	0.723			
Others	11	37.9	18	62.1	1.81	0.82–4.01	0.144			
Having problems on medical expenses										
Yes	18	22.2	63	77.8	1.00					
No	90	26.6	249	73.4	1.27	0.71–2.25	0.424			
BMI										
Normal weight	65	27.3	173	72.7	1.00					
Underweight	7	8.9	72	91.1	0.26	0.11–0.59	0.001*			
Overweight	36	35.0	67	65.0	1.43	0.87–2.35	0.157			
Type of medical insurance										
Medical insurance for employees	47	37.0	80	63.0	2.23	1.41–3.53	0.001*			
Basic medical insurance	61	20.8	232	79.2	1.00					
Having comorbidities										
No	43	38.4	69	61.6	3.58	1.54–8.32	0.003*			
Having 1–2 diseases	57	22.4	197	77.6	1.66	0.74–3.73	0.216			
Having 3–4 diseases	8	14.8	46	85.2	1.00					
Types of comorbidities										
Hypertension										
Yes	38	18.2	163	81.1	1.00					
No	70	32.0	149	68.0	2.02	1.28–3.17	0.002*			
Diabetes										
Yes	19	16.1	99	83.9	1.00					
No	89	29.5	213	70.5	2.18	1.26–3.77	0.006*			
Osteoporosis										
Yes	4	7.6	49	92.4	1.00					
No	104	28.3	263	71.7	4.84	1.71–13.76	0.003*			
Others										
Yes	79	29.9	185	70.1	1.00					
No	29	28.6	127	81.4	0.54	0.33–0.87	0.011*			
Current cigarette smoking behaviour										
Current smoker	41	53.3	36	46.7	3.62	2.04–6.40	< 0.001*			
Ex-smoker	27	15.3	149	84.7	0.58	0.33–0.99	0.046*			
Non-smoker	40	24.0	127	76.0	1.00					
Duration of smoking (months)										
< 60	7	63.6	4	36.4	7.00	1.92–25.59	0.003*			
61–120	4	57.1	3	42.9	5.33	1.13–25.21	0.035*			
121–360	29	30.5	66	69.5	1.76	0.96–3.21	0.066			
> 360	28	20.0	112	80.0	1.00					
Number of cigarettes smoked per day										
< 15	65	28.1	166	71.9	1.22	0.55–2.72	0.63			
15–30	34	22.4	118	77.6	0.90	0.39–2.08	0.799			
> 30	9	24.3	28	75.7	1.00					
Area of residence										
Rural	60	30.0	140	70.0	0.65	0.42–1.01	0.056			
Urban	48	21.8	172	78.2	1.00					
Distance from residence to hospital (km)										
< 10	49	21.1	183	78.9	1.00					
≥ 10	59	31.4	129	68.6	1.71	1.09–2.65	0.017*			
A person who took to a hospital										
Oneself	62	30.5	141	69.5	2.05	0.60–7.40	0.272			
Family members	43	21.5	157	78.5	1.28	0.35–4.65	0.71			
Others	3	17.6	14	82.4	1.00					
Current living situation										
Live alone	66	28.0	170	72.0	1.00					
Live with others	42	22.8	142	77.2	1.31	0.84–2.05	0.232			
Exposed to second-hand smoke										
Yes	33	19.4	137	80.6	1.00					
No	75	30.0	175	70.0	1.78	1.12–2.84	0.015*			
Airflow limitation severity										
Mild and moderate	104	49.1	108	50.9	49.11	17.61–136.95	< 0.001*	5.27	1.61–17.26	0.006*
Severe and very severe	4	1.9	204	98.1	1.00			1.00		
Duration of illness (months)										
< 60	94	58.8	66	41.2	78.33	23.96–156.05	< 0.001*	5.57	1.40–22.12	0.015*
60–120	11	12.0	81	88.0	7.47	2.21–27.52	0.003*	0.99	0.22–4.45	0.990
> 120	3	1.8	165	98.2	1.00			1.00		
Home oxygen therapy										
Do not use	91	48.4	97	51.6	62.86	8.55–462.2	< 0.001*			
Short term home oxygen therapy	16	9.8	148	90.2	7.24	0.94–55.75	0.057			
Long-term home oxygen therapy (> 15 h/day)	1	1.5	67	98.5	1.00					
Numbers of hospitalisations within past 3 months										
0	102	47.7	112	52.3	55.1	13.28–228.59	< 0.001*	9.39	1.62–54.43	0.013*
1	4	4.8	79	95.2	3.06	0.55–17.12	0.202	1.47	0.19–11.45	0.716
> 2	2	1.6	121	98.4	1.00			1.00		
Doctor visits within past 3 months										
< 5	79	50.6	77	49.4	103.62	14.01–761.48	< 0.001*			
5–10	28	17.3	134	82.7	21.10	2.82–157.72	0.003*			
> 10	1	1.0	101	99.0	1.00					

*Significance level set at α = 0.05.

In the multivariable analysis, six variables were found to be associated with good QOL. Patients who were employed had 2.35 times (95% CI 1.03–5.34) greater odds of having good QOL than those who were unemployed. Those whose family income was higher than 1,00,000 CNY had 2.49 times (95% CI 1.15–5.39) greater odds of having good QOL than those whose family income was lower than 1,00,000 CNY. Those who had treatment expenses of less than 5000 CNY had 4.57 times (95% CI 1.57–13.30) greater odds of having good QOL than those who had treatment expenses of 5000 CNY or higher. Those who had mild or moderate airflow limitation had 5.27 times (95% CI 1.61–17.26) greater odds of having good QOL than those who had severe or very severe airflow limitation. Those who had a duration of illness less than 60 months had 5.57 times (95% CI 1.40–22.12) greater odds of having good QOL than those who had a duration of illness of 120 months or more. Those who had not been hospitalized within the past 3 months had 9.39 times (95% CI 1.62–54.43) greater odds of having good QOL than those who were hospitalized more than twice over the past 3 months (Table 4).

## Discussion

Only one-third (24.8%) of the COPD patients who lived in Zhejiang Province, Chinn had poor QOL. Several factors were detected as contributors to having good QOL among COPD including employment status, high income, having been charged low treatment fees, having mild and moderated airflow limitation, having been diagnosed with COPD less than 60 months, and having never been admitted in a hospital.

The majority of people living in Zhejiang Province, China, had an annual family income of $14,750, which was higher than that of people living in other regions of China. COPD patients living in Shandong Province, where people had an annual family income lower than that in Zhejiang, had a lower proportion of good QOL^25,31^. Those people who lived in higher-income areas had higher levels of health insurance coverage, which supported them in accessing medical care and having better QOL than those who lived in poorer areas and had lower levels of health insurance coverage^25,31^. This finding confirmed that COPD patients living in high-income areas have better QOL. This means that those who have a higher income would have a better opportunity for early diagnosis, treatment, and continuous care. Once carefully and continuously cared for, patients would have better QOL and less opportunity to be hospitalized due to poor management of the disease. Moreover, we found that COPD patients who were employed had better QOL than those who were not employed. Kupcewicz et al.^32^ reported that COPD patients who were employed had better QOL than those who were retired. However, a large proportion of COPD patients were not actively working^33,34^. Due to its pathogenesis, the disease and the burden of medical expenses could be key impact factors of QOL as well^33^.

Even in our study, smoking was not found to be associated with QOL among COPD patients. However, many studies^35–37^ have reported that smoking is a key factor of poor QOL among COPD patients. Smoking was reported as a significant risk factor for hospitalization among COPD patients^38,39^, especially among COPD patients with poor economic status^39^. A study in Korea^40,41^ reported that COPD patients with a low family income had a greater chance of using cigarettes and had poorer QOL. This could reflect that COPD patients with a poor economic status have a greater chance of stress and start smoking, followed by a severe stage of COPD and low QOL.

Medical expenses or the cost of treatment was a significant factor associated with QOL among COPD patients living in Zhejiang Province, China. Medications account for the highest proportion of total medical costs for COPD patients^42,43^. Zhu et al.^14^ and Li et al.^44^ reported that a high medical cost was a direct factor in reducing QOL among COPD patients. Unaffordable medical costs of COPD patients were associated with a poor stage of COPD and poor QOL^14^. Basically, COPD patients need to attend a hospital regularly to check their health and obtain medications throughout their lives. If a patient cannot pay for medication, they enter a poor stage of the disease and have difficulty breathing, which directly impacts their QOL. Thus, affordable medical care is a significant factor in good QOL among COPD patients.

Our study clearly showed that the severity of airflow limitation among COPD patients was associated with their QOL. It is well known that impairment of lung function leads to a reduction in patients’ ability to carry out daily activities^2,45^. COPD patients with severe airflow limitation often experience dyspnea, cough, fatigue, and declining lung function^46,47^. This affects their participation in social activities, including the limitation of occupational opportunities and interactions with their family members and other social activities. This could develop the individual’s perception of being a burden to others because they need assistance to complete daily activities and finally manifest as impaired QOL^48^. Several studies^49,50^ reported that COPD patients had poor QOL due to personal perceptions of their family members’ burden, especially in the mental health domain.

We found that a longer course of disease led to a poorer level of QOL among COPD patients. On the other hand, those who had a shorter period of COPD development had better QOL than those who had a longer COPD diagnosis. Jankowska-Polańska et al.^51^ also reported that COPD patients who lived for a shorter duration with the disease had a better QOL than those who had lived longer with the disease. Divo et al.^50^ reported that COPD patients who had lived with the disease longer had a greater opportunity to have a heavy cough in daily life than those who had lived with COPD for a shorter time. The study^52^ also reported that coughing was a major sign associated with the QOL of COPD patients. Patients diagnosed over a longer period had a greater chance of being hospitalized than those diagnosed over a shorter period^52^. Several studies^53–56^ reported that COPD patients who had been hospitalized presented panic or mental health problems compared with those who did not, eventually resulting in poorer QOL. Patients with longer illness could face a severe decline in mental health due to the stage of pathogenesis, lack of social interaction, and poorer self-confidence, resulting in poor QOL.

A greater number of hospitalizations indicated disease severity and patients with repeated admissions had significantly reduced QOL. Many studies^57–59^ reported that COPD patients who had been hospitalized had poorer QOL. Physical, psychological, and social life impacts were detected among COPD patients who were frequently admitted to a hospital^60–62^, which directly impacted QOL. Some studies showed that patients with frequent exacerbations had a significantly lower QOL than patients with less frequent exacerbations^63^. Bernhard et al.^64^ reported that changes in HRQOL were more dependent on the frequency of exacerbation than on FEV1 and DLCO decline. Hospitalization also increased the financial burden and reduced QOL^65^. Hospitalization among COPD patients could reduce their QOL due to physical, psychological, social life, and economic reasons.

Some limitations were found in this study that could impact the results and interpretations. First, with the nature of a cross-sectional study that assesses both exposures and outcomes at the same time, quality of life might not be the exact consequence of the preceding factors. Good QOL among individuals might be the integrated outcome of many factors, especially living environment and family relations, which were not measured in our study. Second, the size of the study sample obtained from the standard formula for a cross-sectional study might impact the generalizability of the results to the general population. Last, using telephone calls to collect data might impact the completeness of the data and the quality of the data because physical body language could not be evaluated.

## Conclusion

A large proportion of COPD patients living in Zhejiang Province, China, suffer from poor QOL. Several personal traits and the unaffordable cost of treatment are the major factors contributing to poor QOL among COPD patients. To improve QOL among COPD patients, public health policy-makers must develop a proper channel to increase accessibility to health care services, including affordable health insurance. Health institutes must consider supportive ways to provide medical services for COPD patients. Implementing measures to help COPD patients obtain a better job and higher income for family members is one of the challenges to ensure that COPD patients will be able to access medical care and have good QOL.

## Data Availability

The datasets used and/or analysed during the current study are available from the corresponding author on reasonable request.

## Abbreviations

COPD: Chronic obstructive pulmonary disease
QOL: Quality of life
SGRQ: St. George's Respiratory Questionnaire
AOR: Adjusted odds ratio
IOC: Item-objective congruence
COVID-19: Coronavirus disease 2019
BMI: Body mass index
FEV1: Forced expiratory volume in one second
CI: Confidence interval
OR: Odds ratio
